# The embedded documentation of implementation tracking (EDIT): a low-burden approach to supporting implementation documentation in health research

**DOI:** 10.3389/frhs.2026.1855898

**Published:** 2026-08-12

**Authors:** Alexis Zollo, Rebecca F. Hamm, Meghan B. Lane-Fall, Laura Ellen Ashcraft

**Affiliations:** 1 Department of Obstetrics and Gynecology, University of Pennsylvania, Perelman School of Medicine, Philadelphia, PA, United States; 2 AMETHIST@Penn – Achieving Maternal Empowerment and Transforming Health Through Implementation Science and Training, University of Pennsylvania, Philadelphia, PA, United States; 3 Penn Implementation Science Center, University of Pennsylvania, Philadelphia, PA, United States; 4 Leonard Davis Institute of Health Economics, University of Pennsylvania, Philadelphia, PA, United States; 5 Department of Anesthesiology, Columbia University, New York, NY, United States; 6 Department of Biostatistics, Epidemiology, and Informatics, University of Pennsylvania, Perelman School of Medicine, Philadelphia, PA, United States

**Keywords:** context, implementation science, implementation science (MeSH), pragmatic, tools

## Abstract

**Introduction:**

Effectiveness studies often generate rich insights about implementation processes and context. However, formal implementation evaluation is often challenging to incorporate for many reasons, including limited staffing, competing study priorities, lack of implementation expertise, or the absence of dedicated funding. As a result, relevant implementation information is inconsistently documented or lost altogether. This paper describes the development and early application of the Embedded Documentation of Implementation Tracking (EDIT) tool, a pragmatic instrument designed to facilitate low-burden documentation of implementation information within innovation research.

**Methods:**

EDIT was created through an iterative, theory-informed process drawing on implementation science frameworks and investigator experience. The tool was refined through internal review and four pilot test cases use across multiple research contexts, including both NIH and VA-funded work, and multiple study types including, innovation effectiveness and implementation-effectiveness hybrid studies. Written feedback was solicited from participating teams regarding clarity, feasibility, and utility.

**Results:**

Feedback from four research teams representing four projects indicated that EDIT was understandable, practical to use during routine research activities, and helpful for capturing implementation observations without increasing workload. Users reported that the structured, yet adaptable format facilitated reflection on adaptations, determinants, contextual influences, and implementation strategies, particularly in early-stage or resource-constrained studies.

**Discussion:**

Embedding documentation within existing meetings and workflow was viewed as a major strength. EDIT provides a feasible strategy for strengthening implementation documentation capacity and producing organized insights that can better contextualize study outcomes.

## Introduction

Effectiveness and implementation are inseparable. As health innovations are studied, teams are simultaneously selecting strategies, navigating barriers, and adapting their approaches to fit local context. Understanding these implementation processes is critical to interpreting clinical outcomes and to assessing *whether* and *how* an innovation can be replicated at scale (1). Yet many empirical studies are not designed or resourced to conduct formal implementation evaluations. As a result, teams often fail to document the information needed to understand implementation: what factors are facilitating or impeding delivery, how and why innovations are being adapted, and how context shapes outcomes across settings. This information is frequently discussed informally or lost altogether, limiting opportunities to learn from both successes and failures (2, 3).

This challenge is particularly salient for early career investigators and research teams for whom implementation science was not incorporated into the original study design. Traditional data collection in implementation science methods such as interviews and surveys require time and financial resources that may simply be unavailable (4). Without accessible infrastructure for documentation, teams who lack dedicated implementation science capacity cannot contribute to, or benefit from, the growing knowledge that depends on this information.

There is growing recognition that pragmatic tools can help bridge this gap. In implementation science, pragmatic tools are those designed to minimize burden, integrate into existing workflows, and produce actionable information without requiring specialized training or resources (5, 6). Their value is not simply that they are easier to use, but that they make the incorporation of implementation science possible for teams that would otherwise be excluded entirely. Pragmatic tools serve a function distinct from formal evaluation instruments. Rather than producing psychometrically precise measurement, they support structured reflection, facilitate shared understanding within teams, and generate documentation that may later be analyzed with more rigorous methods (6, 7). The field of implementation science has acknowledged an inherent tension between pragmatism and rigor (8). This prompts a necessary diversification of methods to reach teams and settings that would otherwise remain outside of implementation science.

Prior efforts to support implementation documentation focus on discrete domains of the implementation process. Some teams used activity logs or meeting-based tracking approaches to capture implementation strategies (9, 10). Others have applied adaptation-focused frameworks such as Framework for Reporting Adaptations and Modifications to Evidence-Based Interventions (FRAME) to classify modifications to evidence-based interventions (11). Moreover, many approaches and tools for documentation are designed for those with familiarity with implementation science concepts. For example, Map2Adapt focuses on planning for adaptations, FRAME-IS addresses implementation strategies, and TIdieR supports *post-hoc* descriptions of interventions for replication and publication (12–14). While each tool has utility within its intended scope, meaningful use requires prior familiarity with implementation science concepts, a prerequisite that places them outside the reach of many research teams. A single, structured instrument capable of capturing implementation determinants, strategies, adaptations, contextual factors, and community engagement without requiring that prior knowledge remains underdeveloped.

We developed the Embedded Implementation Tracking tool (EDIT) with this purpose in mind. EDIT is a low-burden, semi-structured tool that captures implementation-related information: the factors, processes, and adaptations that shape how an innovation is delivered in practice. This includes barriers and facilitators, strategies, contextual influences, and community engagement. The domains included in EDIT were chosen because they represent the core implementation information necessary for most implementation research and map onto constructs recognized across major implementation frameworks, including the Consolidated Framework for Implementation Research (CFIR) and the Exploration, Preparation, Implementation, Sustainment framework (EPIS) (15–17). CFIR is a determinant framework that identifies the multilevel factors, spanning inner organizational context, outer setting, and innovation characteristics, that facilitate or impede implementation (17). EPIS functions as both a stage-based process model, defining four phases from exploration through sustainment, and a contextual framework describing how inner and outer factors shape implementation across those phases (16).

EDIT does not operationalize these constructs for formal measurement; rather, it uses them as organizing categories to prompt structured reflection during routine study activities, team meetings, interested party discussions, and similar touchpoints where implementation-relevant information naturally surfaces but is rarely captured. Importantly, EDIT is designed to be completed by any engaged team member present at these meetings. No prior implementation science training is required; familiarity with the innovation or the local context is sufficient for most domains, and capturing multiple perspectives within a team can further strengthen documentation quality. This paper describes the development and early use of EDIT and presents initial feedback on its clarity, feasibility, and perceived utility as an infrastructure tool for implementation science across resource contexts.

## Methods

### Context

This work was conducted within the Implementing a Maternal health and PRegnancy Outcomes Vision for Everyone (IMPROVE) Initiative and led by AMETHIST@Penn (18). IMPROVE is a National Institutes of Health effort to reduce preventable causes of maternal mortality and improve health for women before, during, and after pregnancy, with a special emphasis on populations who are disproportionately affected. The IMPROVE initiative funds individual grants and 12 Centers of Excellence, each encompassing one to three individual research projects, representing a diverse range of geographies, populations, and study designs, from observational studies to multi-site randomized trials. The University of Pennsylvania established the Achieving Maternal Empowerment and Transforming Health through Implementation Science and Training (AMETHIST) Hub to support NIH IMPROVE by supporting research teams through the process of implementation research, accelerating implementation skills development, and advancing knowledge about successfully conducting implementation research.

Across the Network, many of these trials were not originally designed as formal implementation trials or with collection of implementation data in mind. As a result, implementation-relevant insights were often generated but not captured. We sought to identify low-burden ways to document latent implementation factors often discussed in routine research meetings as an opportunity for data capture. We developed EDIT as a pragmatic tool to embed implementation tracking within existing research workflows. Routine team meetings were already surfacing implementation-relevant observations; EDIT provided a structure to capture them.

### Initial development

EDIT was developed through an iterative process grounded in investigator experience conducting implementation evaluations. The development team included investigators with expertise in maternal health, health services research, methodology, and implementation science, with a shared interest in pragmatic approaches to advancing implementation science capacity. The constructs captured in EDIT were selected based on their prevalence across commonly used implementation frameworks and their practical relevance to the types of discussions already occurring in research team meetings. Specifically, the domains of implementation determinants, strategies, and contextual factors reflect constructs central to CFIR, particularly its inner and outer setting domains and intervention characteristics (17). The adaptation domain draws on FRAME (11).

Documentation of adaptations enables teams to understand how and why an innovation is changing over time, while tracking determinants surfaces the conditions enabling or impeding delivery. Capturing strategies provides a record of what teams are doing in response to implementation challenges, and recording context situates findings within the specific circumstances of a setting. Documenting community and stakeholder engagement reflects the relational dimensions of implementation that influence uptake. Together, these domains create a structured record that supports both real-time decision-making and retrospective interpretation of outcomes.

Importantly, EDIT was not designed to operationalize or formally measure these constructs. Rather, existing implementation science frameworks informed the identification of content areas most likely to yield meaningful documentation during routine research activities. Early drafts emphasized simplicity, minimal burden, and adaptability across study designs and settings. AMETHIST@Penn investigators reviewed draft versions of EDIT internally. EDIT was then piloted across multiple research contexts, including effectiveness studies, hybrid trials, and quality improvement projects. Pilot use occurred during standing research meetings and routine project activities, without requiring additional training or dedicated data collection sessions. Feedback focused on clarity of prompts, redundancy across domains, and feasibility of completion during routine meetings. The team revised the tool to streamline language, reduce duplication, and clarify the intended scope of each domain.

### Feedback

Four research teams piloted EDIT over a period of approximately 4 months from March 2025 through June 2025. Teams were selected as a convenience sample of AMETHIST-affiliated projects that were actively engaged in research meetings during the development period and had not been involved in the tool's initial design, minimizing familiarity bias. The four projects represented a range of study designs, settings, and populations: two IMPROVE Centers of Excellence focused on maternal health equity, an implementation science study of clinical handoff processes within an academic medical center, and a national Veteran's Affairs quality improvement project supporting the scale-up of evidence-based practices across the United States. These teams were identified as relevant test cases because they varied in their implementation science capacity: two were led by investigators with formal implementation science expertise, while two had limited prior exposure to implementation science and no dedicated implementation evaluation staff. This variation was informative, as it allowed for feedback on EDIT's utility and accessibility across different levels of implementation capacity.

Following approximately 1 month of use, the principal investigator (AZ) contacted the lead of each participating team via email to solicit open-ended feedback on their experience. Teams were asked to reflect on what worked, what felt less relevant, and what may have been missing. No structured feedback instrument was used. Teams completed EDIT entries as part of their routine meeting activities and did not conduct formal analysis of their own documentation; feedback reflected their experience of using the tool rather than analysis of the content it produced. Responses were solicited through an email prompt and reviewed descriptively by AZ and LEA to identify recurring themes and inform revisions to wording and structure. This feedback was not intended as formal tool validation, but rather as early input to guide iterative development.

## Results

Across teams, feedback was largely consistent. First, the tool was understandable without prior implementation science training. Second, it was feasible to complete during or immediately after standing meetings. Lastly, it provided useful structure for organizing observations that would otherwise be discussed informally or not at all. One respondent described the tool as “a nice moment of reflection post-retreat,” noting that it was “easy to fill out, not too long,” and helped identify “next steps…to keep momentum going.” Another highlighted its function for team cohesion, stating that the form was “wonderful to track changes and keep the team on the same page, especially for those who missed the meeting.”

Suggestions for enhancement included expanding prompts related to community engagement, clarifying the role of observer reflections, and adding optional structural features such as a shared platform format or completion date column. Minor revisions were made to streamline language, clarify domain boundaries, reinforce flexibility across settings, and incorporate guidance for real-time vs. post-meeting completion. While feedback supported the acceptability and usability of EDIT, the primary outcome of this work is the finalized tool itself.

### The final EDIT tool

EDIT is a semi-structured meeting-based template designed to support documentation of implementation-related information during routine research activities. The tool is intended to be completed during or shortly after standing meetings in which implementation issues naturally arise, such as evaluation team check-ins, project management meetings, or community partner discussions. Several specific design features support these goals.

First, the tool is organized to mirror the natural flow of a research team meeting. It opens with logistical and contextual information (who was present, what was discussed), moves through substantive implementation domains, and closes with follow-up actions and observer reflections. This structure allows users to complete the tool in sequence during the meeting or immediately after it concludes, without requiring complete reconstruction from memory. Second, each domain uses open-ended, plain language prompts rather than Likert-scale ratings or coded categories, allowing users to document what is relevant to their context without forcing observations into predetermined structures. Third, users are explicitly instructed to complete only the domains relevant to a given meeting, reducing the burden of partial or sparse entries and reinforcing that incomplete documentation is still valuable. The finalized version of the tool is provided as a Microsoft Word document (see Supplementary File 1 for the complete blank template). Table 1 illustrates the Meeting and Contextual Information section; Tables 2–6 present the remaining domains with a completed case example.

**Table 1:** Meeting and contextual information.

Meeting name	CHW Program Implementation Team Check-In
Topic of meeting	Progress Review: Postpartum Follow-Up CHW Program
Date	February 4th, 2026
Time	10:00 AM EST
Location	Zoom
Site name	Site A (Urban Academic Medical Center), Site B (Federally Qualified Health Center)
Participants names/roles	Dr. Rivera (PI), Dr. Lee (Co-PI), Nurse Taylor (Site A Coordinator), Dr. Patel (Site B Lead), Research Assistant Jordan
Facilitator	Dr. Rivera

**Table 2:** Intervention information.

Intervention *Evidence-based practice, “the thing”, etc.*	
What is the intervention?	CHW postpartum follow-up program Trained CHWs provide structured outreach, navigation, and support to Black birthing people at high risk for postpartum hypertension. CHWs conduct discharge planning, coordinate follow-up appointments, provide blood pressure monitoring education, and connect patients with social resources.
Describe any changes made to the intervention itself, the people conducting the intervention, the people receiving the intervention?	Site B adapted the home visit protocol to telehealth due to transportation barriers and patient preference Blood pressure monitoring instructions simplified at Site B after CHWs reported patients had difficulty with written materials Additional check-in call added at two-weeks postpartum at Site A in response to patient feedback about feeling unsupported between scheduled visits

**Table 3:** Implementation determinants.

Implementation determinants *Factors that influence the ability to implement the intervention*	
What has been getting in the way of implementing the program?	Patients at Site B frequently miss postpartum appointments due to lack of transportation and childcare CHWs at Site A report difficulty reaching patients by phone after discharge Limited clinical buy-in from some providers at Site B who are unfamiliar with the CHW role
Who has raised these concerns?	CHWs at both sites Site B clinical lead and nursing staff Research coordinators tracking postpartum contact rates
What patterns if any have you observed in determinants?	Transportation and social support barriers are more pronounced at Site B Provider unfamiliarity with CHW scope of practice is a recurring theme at Site B and appears to be limiting referral uptake
What strength(s) exist within the setting related to the implementation?	Strong CHW workforce infrastructure and supervisory support at Site A Site B leadership has expressed commitment to reducing racial disparities in maternal outcomes and is actively problem-solving barriers
How are communities overcoming/engaging with these determinants?	Site B Chws Are Accompanying Patients To Appointments When Possible And Connecting Patients To Local Transportation Resources Site A CHW Supervisor Is Developing A Brief Orientation For Clinical Staff On CHW Roles And How To Make Referrals

**Table 4:** Implementation strategies.

Implementation strategies *What we do to help our target population to receive the intervention*	
What approaches have been used?	CHW-led outreach calls initiated within 48 h of discharge Monthly team huddles at each site to review caseloads and problem-solve barriers Academic detailing sessions with Site B providers to build familiarity with CHW program and referral process
Who is using them?	CHWs at both sites conducting outreach and follow-up Research team facilitating huddles and coordinating cross-site learning Site A CHW supervisor leading academic detailing at Site B
What problems, concerns, or clear gaps are present related to these strategies?	Monthly huddles are inconsistently attended at Site B due to staffing constraints Academic detailing has reached nursing staff but not yet OB/GYN attendings at Site B No formal strategy yet in place to address patient-level transportation barriers beyond informal CHW support

**Table 5:** Community partners and contextual information.

Engagement with community partners	
Which community partners have been involved in identifying barriers/solutions? Describe	*Local Black maternal health advocacy organization has been engaged at Site B to help identify culturally appropriate outreach messaging and connect patients with local resources* *A community advisory board including past program participants has provided input on communication preferences and barriers to postpartum follow-up*
*Context*	
What situational or environmental factors were influencing the discussions?	*State and county health departments have recently issued updated guidance on postpartum care visits for Medicaid-insured patients, which may affect reimbursement structures at Site B. The research team is monitoring whether these policy changes will affect the feasibility of the current visit schedule and whether additional documentation will be required for program sustainability."*

**Table 6:** Follow-up actions and observer reflections.

Follow-up actions	
What follow-up tasks were assigned?	*Research coordinator to pull 30-day postpartum contact rates by site and share at next meeting* *Site B clinical lead to identify two OB/GYN attendings for next academic detailing session* *Dr. Rivera to follow up with transportation assistance program about potential partnership for Site B patients*
Observer reflections	
What stood out most about this discussion?	*Site B is making meaningful progress despite resource constraints; the community advisory board engagement appears to be building trust with the patient population in ways that are beginning to improve contact rates*
Describe any notable dynamics or trends.	*There is a growing sense of shared ownership between the research team and Site B clinical staff that was not present at earlier meetings—worth monitoring as a potential facilitator of implementation* *CHW burnout was raised informally after the meeting ended and should be addressed explicitly at the next check-in*

#### Case example context

The following example is drawn from an effectiveness trial that was not originally designed to include formal implementation evaluation. A team is implementing a community health worker program to improve postpartum follow-up care among Black birthing people at high risk for postpartum hypertension. The program pairs trained community health workers (CHWs) with patients at two sites that differ substantially in resources, infrastructure, and prior CHW experience. Site A is an urban academic medical center with an established CHW workforce and existing postpartum care infrastructure, whereas Site B is a federally qualified health center serving predominantly rural, Medicaid-insured population with limited prior experience with CHW programs. The team uses EDIT to document implementation processes as the program moves from early launch into active delivery, focusing on capturing site-specific adaptations and emerging barriers before they are lost to informal discussion. The individuals, sites, and circumstances described are hypothetical and are not drawn from any specific study or participant. This case example illustrates how EDIT can be used in a multi-site, resource-variable context.

### Core domains of the EDIT

#### Meeting and contextual information

The tool begins with descriptive fields, including meeting name, topic, date, location, site, participants, and meeting facilitator. These fields allow entries to be linked to specific activities, sites, and time points. Each meeting generates a new EDIT entry with the same domain headings and metadata field, allowing users to review entries sequentially to identify patterns over time. In practice, teams are encouraged to maintain a running EDIT log in one place, whether as a multi-tab document, a sequentially appended Word file, or within a shared folder. This allows entries to accumulate and be reviewed before subsequent meetings or during analysis.

#### Phase of implementation

The phases of implementation (e.g., planning, early implementation, active implementation, sustainment) are drawn from stage-based implementation frameworks, such as EPIS (16). Situating observations within a phase is important because different challenges, strategies, and adaptations become relevant at different points in the implementation process. An entry documenting high rates of provider resistance means something different in early implementation than in sustainment. Users are prompted to identify the current phase of implementation. This field situates observations temporally and supports interpretation of implementation challenges and adaptations over time.

EPIS functions as both a stage-based process model organizing the sequence of implementation and a contextual framework describing the inner and outer factors that shape implementation across those phases (16). To support users who may be unfamiliar with implementation phases, the tool includes brief illustrative descriptors alongside each phase label. For example: planning (pre-launch; finalizing protocols and training); early implementation (first uses; identifying initial barriers and making early adjustments); active implementation (ongoing delivery, refining strategies and monitoring fidelity); and sustainment (maintaining delivery over time; planning for scale). These descriptors are designed to allow users to self-identify their phase without requiring prior knowledge of implementation science terminology.

#### Innovation and adaptations

Documenting the innovation itself and any modifications made during delivery is foundational to implementation research. Without a clear record of what was delivered, and how it changed over time, teams cannot assess fidelity, interpret variation in outcomes across sites, or provide the replication information necessary for scale-up. This domain draws on FRAME, which provides a taxonomy for classifying the nature, reason, and impact of modifications to evidence-based interventions (11). This section describes the innovation being implemented and to document any changes made to the innovation itself, the individuals delivering the innovation, or the populations receiving it. The emphasis is on capturing adaptations as they emerge, rather than categorizing or evaluating them.

#### Implementation determinants

The Determinants section is designed to capture contextual factors influencing implementation, including barriers and facilitators. Prompts encourage users to note what has been getting in the way of implementation, who has raised specific concerns, observable patterns in determinants, and strengths within the setting that support implementation.

Tracking barriers and who has raised them serves three purposes: it identifies whether barriers are concentrated among particular stakeholder groups (e.g., frontline staff vs. leadership), whether concerns are being surfaced broadly or by a limited subset of voices, and supports more targeted strategy development.

Tracking how determinants are being overcome is distinct from broader implementation strategies. The determinants domain focuses on organic, often informal, or community-initiated responses to barriers or actions that emerge from the context itself rather than from deliberate strategy deployment by the research or implementation team. By contrast, the strategies domain captures deliberate, team-initiated actions designed to support implementation (19). For example, a CHW informally accompanying a patient to an appointment is captured under Determinants as a contextual response to a transportation barrier; a formal team decision to implement a transportation assistance partnership is captured under Strategies. Teams are encouraged to use their judgment in placement and to note any actions documented in both sections for cross-reference. It should be noted that teams conducting effectiveness research without a formal implementation science framework may find the Determinants domain more immediately applicable, as deliberate implementation strategies may not yet be in use or may emerge organically rather than by design.

#### Implementation strategies

This domain focuses on strategies used to support innovation delivery or address implementation challenges. Users document what approaches have been used, who is using them, and any problems, gaps, or concerns related to these strategies. This section allows teams to track implementation efforts over time and to reflect on alignment between challenges and responses.

The “problems, concerns, and gaps related to strategies” prompt captures issues with the strategies themselves, whether a strategy is being executed as intended, whether it is reaching the right people, or whether there are logistical barriers to its consistent use. This is distinct from implementation determinants, which refer to the underlying contextual factors shaping implementation broadly. A strategy may be well-designed but poorly executed; documenting concerns about strategies allows teams to distinguish between implementation failures due to contextual barriers and those due to strategy design or delivery problems. For teams without formal implementation strategies, this section can be used to document any informal or emergent approaches that have developed during the study.

#### Context and community partner engagement

Community and stakeholder engagement is not peripheral to implementation; it shapes the conditions under which an innovation can take hold. CFIR's outer setting constructs explicitly encompass the external environment in which implementation occurs, including the needs and resources of the broader community and the role of external stakeholders in influencing uptake (17). Documenting engagement with community partners captures the relational and contextual dimensions of implementation that formal strategy tracking often misses, and surfaces how local knowledge and community-identified priorities are informing implementation decisions in real time. Separate sections prompt users to document situational or environmental factors influencing implementation discussions, as well as engagement with community or stakeholder partners in identifying barriers or solutions. These domains emphasize the role of context and partnership in shaping implementation processes.

#### Follow-up actions and observer reflections

The final sections capture follow-up tasks assigned during the meeting and allow space for observer reflections. Observer reflections are designed to capture the qualitative texture of implementation discussions that does not fit neatly into structured domains: emerging team dynamics, shifts in engagement or morale, or observations about the implementation climate. These observations are important for interpretation because implementation outcomes are shaped not only by formal strategies and identifiable barriers, but by relational and cultural factors that are often only perceptible to those present in the room.

### Analytic and operational uses of EDIT documentation

Information captured using EDIT is intended to support multiple analytic and operational functions. Teams can use documented observations to track adaptations over time, identify emerging determinants of implementation, and make real-time adjustments to strategies or workflows. The accumulated record also provides structured context for interpreting variation in innovation delivery and outcomes, strengthening transparency in reporting and facilitating cross-project learning. In addition, systematically organized documentation can inform replication, scale-up planning, and future grant development by clarifying what conditions were necessary for implementation success and what challenges required modification.

The analytic purpose of EDIT documentation will vary depending on study design, stage of implementation, and available resources. In formative applications, accumulated entries can support real-time course correction: identifying barriers, tracking whether strategies are being executed as intended, and surfacing adaptation patterns before they are lost to informal discussion. In summative applications, the record produced by EDIT can contribute to implementation reporting, support construction of adaptation logs, and provide the contextual narrative necessary for interpreting variation in outcomes across sites or time points. These uses are not mutually exclusive; the same documentation may serve both purposes within a single study. The specific analytic approaches best suited to EDIT-generated data, whether qualitative content analysis, framework-based analysis, or other methods, will depend on the research question and available capacity, and represent an important direction for future investigators.

## Discussion

This paper describes the development and early application of EDIT, a pragmatic, meeting-embedded tool designed to support low-burden documentation of implementation-related information in health innovation research. Pilot feedback from four research teams across varied study designs and resource contexts indicated that EDIT was understandable, feasible, and useful for capturing implementation observations without adding meaningful burden to routine workflows. EDIT is designed specifically for teams whose original study design did not include formal implementation evaluation, providing an entry point into implementation science that can support more structured analysis as capacity grows.

There is an established need for pragmatic and accessible tools in implementation science (5, 6, 15). EDIT's design reflects the principles of pragmatic tool development: brevity, embeddedness in existing workflows, and flexibility across contexts (5, 6). EDIT was developed with the considerations identified by Martinez and colleagues in mind: grounding domains in recognized implementation constructs, minimizing burden on users, and matching the tool's purpose to its use case (7). EDIT's purpose is documentation and facilitation, not formal measurement (7). EDIT deliberately departs from traditional instrumentation standards. It lacks reliability testing and formal validation by design, consistent with its role as a pragmatic infrastructure tool rather than a psychometric instrument (7).

A preliminary alignment between EDIT's domains and established implementation frameworks clarifies this grounding. The Determinants domain maps onto the inner and outer setting constructs of CFIR, capturing both organizational characteristics and broader contextual factors that shape implementation (17). The Strategies domain aligns with the active implementation and implementation support components of EPIS (16). EPIS also informed the Phases domain, where its stage-based process model provides the organizational structure for situation observations across the implementation continuum (16).

EDIT is not designed for real-time classification of strategies against established taxonomies. Requiring users to apply taxonomic coding during or immediately after meetings would reintroduce the implementation science expertise burden the tool is explicitly designed to avoid. Instead, EDIT captures strategies descriptively as they are discussed, preserving enough detail to support retrospective classification against frameworks if and when the study design, analytic goals, or team capacity warrant it. This positions EDIT as a documentation infrastructure that can feed into more formal strategy analysis rather than replacing it (19).

The Adaptations domain corresponds to FRAME, which provides a taxonomy for classifying modifications to evidence-based interventions (11). Importantly, EDIT does not operationalize these frameworks for formal measurement; rather, this alignment suggests that documentation generated through EDIT can be connected to framework-based analyses when teams develop the capacity or resources to pursue them, creating a potential pathway from pragmatic tracking toward more rigorous evaluation.

EDIT is designed to function both as a complement to established implementation data collection methods and as a standalone option when those methods are unavailable. In settings where formal evaluation is feasible, EDIT provides ongoing documentation that captures implementation processes as they unfold, filling gaps between more intensive data collection events. In settings where formal evaluation is not feasible due to resource constraints, limited staffing, or limited implementation science expertise, EDIT serves as the primary infrastructure for implementation documentation. This dual function is intentional: pragmatic tools should add value across the spectrum of research capacity, not only at its lower end (6).

The analytic utility of this documentation extends beyond individual teams. Accumulated EDIT entries can support the types of implementation-focused analyses increasingly recognized as essential to implementation science: constructing adaptation logs, identifying patterns in implementation determinants across sites, and generating the implementation narrative necessary for transparent reporting (11, 17). As Martinez and colleagues argue, implementation instruments should be designed not only to measure but to facilitate the ongoing work of implementation, a principle EDIT operationalizes through its meeting-embedded format (7). As entries accumulate across meetings and phases, the documentation progressively informs both the conduct of the study and its eventual reporting. This reduces reliance on retrospective recall, which has been identified as a major source of implementation documentation loss (3). Across these applications, EDIT functions as both a data collection instrument and a memory system that makes implicit implementation knowledge explicit and recoverable.

EDIT’s contribution to implementation science lies in lowering the barrier to engaging with implementation concepts for teams that have historically been outside the reach of formal implementation evaluation (8). This is particularly relevant for early-stage studies, under-resourced teams, and investigators new to implementation science. In this way, EDIT can serve as an entry point into implementation science for teams whose original study design did not include implementation evaluation, thus creating documentation that can support more formal analysis as capacity grows. EDIT provides a structured yet adaptable framework for capturing what teams are already observing (determinants, strategies, adaptations, contextual influences). In doing so, it supports the development of implementation literacy and a shared language around implementation processes, while generating documentation that informs the interpretation of outcomes.

### Limitations

Several limitations of this work should be acknowledged. First, the feedback informing EDIT's iterative refinement was gathered from a small number of research teams which may limit the generalizability of findings regarding feasibility and utility. Second, feedback was collected descriptively and was not designed as a formal psychometric evaluation; as such, no claims regarding reliability or validity can be made at this time. Third, the quality and completeness of EDIT entries is likely to vary across users, meetings, and contexts, and this variability was not systematically examined. Documentation quality depends on the consistency and engagement of the individual completing the tool, and entries produced under time pressure or by less experienced team members may capture implementation data less comprehensively. This limitation is inherent to the tool's purpose: EDIT is not designed to produce psychometrically valid data, but to support documentation practices that would not otherwise occur. The value of that documentation, however imperfect, lies in making implementation processes visible and discussable within teams.

### Future directions

Future research should examine the use of EDIT across a broad range of settings, innovation types, and study designs. Additional work could explore how information captured through EDIT aligns with formal implementation frameworks and outcomes, as well as how the tool might be adapted or expanded to support different stages of implementation. Systematic testing of acceptability and utility across contexts may further clarify the role of EDIT as a scalable infrastructure tool within D&I science.

## Conclusion

The implementation knowledge generated within hybrid and effectiveness studies is too valuable to lose, yet without accessible infrastructure to capture it, that knowledge routinely goes undocumented. EDIT addresses this gap by providing a low-burden, flexible tool that embeds implementation documentation within routine research activities. Pilot feedback from four research teams across diverse study types, resource contexts, and implementation stages suggests that EDIT is practical, understandable, and accessible without requiring prior implementation science training or dedicated evaluation infrastructure. While broader validation is needed, this early evidence indicates that the tool's flexible, meeting-embedded format is accessible across the range of settings and study designs represented in the pilot. As the field continues to prioritize pragmatic approaches to implementation research, tools like EDIT may play an important role in ensuring that implementation-related information is systematically preserved. Without such tools, knowledge is routinely lost to informal discussion or retrospective recall.

## Data Availability

The raw data supporting the conclusions of this article will be made available by the authors, without undue reservation.

## References

[1] Bauer MS Kirchner J. Implementation science: what is it and why should I care? Psychiatry Res. (2020) 283:112376. 10.1016/j.psychres.2019.04.025 31036287

[2] Chu KM Weiser TG. Real-world implementation challenges in low-resource settings. Lancet Glob Health. (2021) 9:e1341–2, Elsevier. 10.1016/S2214-109X(21)00310-7 34418381

[3] Stetler CB Legro MW Wallace CM Bowman C Guihan M Hagedorn H. The role of formative evaluation in implementation research and the QUERI experience. J Gen Intern Med. (2006) 21:S1–8. 10.1007/s11606-006-0267-9 16637954 PMC2557128

[4] Brownson RC Graham AC Enola KP, (eds), Dissemination and Implementation Research in Health: Translating Science to Practice, 2nd edn. New York: Oxford Academic (2018) 10.1093/oso/9780190683214.001.0001

[5] Ramanadhan S Revette AC Lee RM Aveling EL. Pragmatic approaches to analyzing qualitative data for implementation science: an introduction. Implement Sci Commun. (2021) 2:70. 10.1186/s43058-021-00174-1 34187595 PMC8243847

[6] Glasgow RE. What does it mean to be pragmatic? Pragmatic methods, measures, and models to facilitate research translation. Health Educ Behav. (2013) 40:257–65. 10.1177/1090198113486805 23709579

[7] Martinez RG Lewis CC Weiner BJ. Instrumentation issues in implementation science. Implement Sci. (2014) 9:118. 10.1186/s13012-014-0118-8 25185799 PMC4164742

[8] Aschbrenner KA Walsh-Bailey C Brown MC Khan T Baggett TP Jones SMW. Practical considerations for engaging staff in resource-constrained healthcare settings in implementation research: a qualitative focus group and consensus building study. J Clin Transl Sci. (2025) 9:e65. 10.1017/cts.2025.29 40201643 PMC11975774

[9] Walsh-Bailey C Palazzo LG Jones SMW Mettert KD Powell BJ Wiltsey Stirman S. A pilot study comparing tools for tracking implementation strategies and treatment adaptations. Implement Res Pract. (2021) 2:26334895211016028. 10.1177/26334895211016028 37090012 PMC9978654

[10] Bunger AC Powell BJ Robertson HA MacDowell H Birken SA Shea C. Tracking implementation strategies: a description of a practical approach and early findings. Health research policy and systems. BioMed Central. (2017) 15(1):15. 10.1186/s12961-017-0175-y PMC5324332 28231801

[11] Wiltsey Stirman S Baumann AA Miller CJ. The FRAME: an expanded framework for reporting adaptations and modifications to evidence-based interventions. Implement Sci. (2019) 14:58. 10.1186/s13012-019-0898-y 31171014 PMC6554895

[12] Hoffmann T Glasziou P Boutron I Milne R Perera R Moher D. [Better reporting of interventions: template for intervention description and replication (TIDieR) checklist and guide]. Gesundheitswesen. (2016) 78:e174. 10.1055/s-0037-1600948 29298449

[13] The Center for Implementation. Map2Adapt tool [Internet]. The Center for Implementation. Available online at: https://thecenterforimplementation.com/map2adapt-tool (Accessed June 29, 2026).

[14] Miller CJ Barnett ML Baumann AA Gutner CA Wiltsey-Stirman S. The FRAME-IS: a framework for documenting modifications to implementation strategies in healthcare. Implement Sci. (2021) 16:36. 10.1186/s13012-021-01105-3 33827716 PMC8024675

[15] Birken SA Powell BJ Shea CM Haines ER Alexis Kirk M Leeman J. Criteria for selecting implementation science theories and frameworks: results from an international survey. Implement Sci. (2017) 12:124. 10.1186/s13012-017-0656-y 29084566 PMC5663064

[16] Moullin JC Sabater-Hernández D Fernandez-Llimos F Benrimoj SI. A systematic review of implementation frameworks of innovations in healthcare and resulting generic implementation framework. Health Res Policy Syst. (2015) 13:16. 10.1186/s12961-015-0005-z 25885055 PMC4364490

[17] Damschroder LJ Reardon CM Widerquist MAO Lowery J. The updated consolidated framework for implementation research based on user feedback. Implement Sci. (2022) 17:1–16. 10.1186/s13012-022-01245-0 36309746 PMC9617234

[18] Amethist. Home | Penn Amethist [Internet]. Available online at: https://amethist.org/ (Accessed June, 29 2026).

[19] Powell BJ Waltz TJ Chinman MJ Damschroder LJ Smith JL Matthieu MM. A refined compilation of implementation strategies: results from the expert recommendations for implementing change (ERIC) project. Implement Sci. (2015) 10:21. 10.1186/s13012-015-0209-1 25889199 PMC4328074

