# Supplementary material for: The embedded documentation of implementation tracking (EDIT): a low-burden approach to supporting implementation documentation in health research
Source: Front Health Serv. 2026 Aug 12;6:1855898. doi: 10.3389/frhs.2026.1855898 (PMC13506754; doi:10.3389/frhs.2026.1855898)
Supplement: Supplementary file 1 [file Supplementaryfile1.docx]

**Introduction**

This form provides a semi-structured approach to documenting observations, challenges, and facilitators that may arise during an implementation study. The goal of this form is to enable teams to gather insights into factors influencing the implementation of their specific intervention without placing additional burden on staff or requiring extensive resources. It may be particularly useful for teams with limited capacity to conduct interviews or surveys but who wish to systematically consider and document key implementation factors.

**Instructions for Use**

**When to Use This Form:**

- During regular activities, such as team meetings or community partner discussions, when implementation factors may naturally arise.
- When resources for traditional data collection methods, such as interviews or surveys, are limited.
- When you want to minimize the workload for your team while still capturing relevant implementation-related data

**How to Use This Form:**

- Identify key moments or activities in your study where implementation-related observations can be recorded (e.g., team meetings, progress updates, stakeholder feedback sessions).
- Use the form to document relevant factors such as barriers, facilitators, contextual influences, and any adaptations made to the intervention or study procedures.
- Ensure that entries are concise but detailed enough to provide meaningful insights.
- This form includes an exhaustive list of potential implementation factors; **not every box needs to be filled out at every meeting**, and some items may not apply to your specific context. Use it as a broad guide to capture what is relevant to your study.

**What to Consider:**

- Tailor the form to align with your study’s specific goals and the nature of the intervention being implemented
- Encourage multiple team members to contribute observations to ensure a comprehensive understanding of the implementation process
- Review the completed forms regularly to identify emerging patterns or trends that may warrant further exploration.

**Meeting Template**

| Meeting Name |  |
| --- | --- |
| Topic of Meeting |  |
| Date |  |
| Time |  |
| Location |  |
| Site Name |  |
| Participants *names/roles* |  |
| Facilitator |  |

| **Discussion Summary** | |
| --- | --- |
| Current Phase of Implementation | |
| What is the current phase of the implementation? | Planning (pre-launch, finalizing protocols and training)  Early implementation (first uses; initial barriers and making early adjustments)  Active implementation (ongoing delivery; refining strategies and monitoring fidelity)  Sustainment (maintaining delivery over time; planning for scale)  Other |
| Intervention  *Evidence-based practice, “the thing”, etc.* | |
| Describe any changes made to the intervention itself, the context, the people conducting the intervention, the people receiving the intervention? |  |
| Implementation Determinants *Factors that influence the ability to implement the intervention* | |
| What has been getting in the way of implementing the program? |  |
| Who has raised these concerns? |  |
| What patterns if any have you observed in determinants? |  |
| What strength(s) exist within the setting related to the implementation? |  |
| How are communities overcoming/engaging with these determinants? |  |
| Implementation Strategies  *What we do to help our target population to receive the intervention* | |
| What approaches have been used? |  |
| Who is using them? |  |
| What problems, concerns, or clear gaps are present related to these strategies? |  |
| Engagement with Community Partners | |
| Which community partners have been involved in identifying barriers/solutions? Describe |  |
| Context | |
| What situational or environmental factors influencing the discussions? |  |
| Follow-Up Actions | |
| What follow-up tasks were assigned? |  |
| Observer Reflections | |
| What stood out most about this discussion? |  |
| Describe any notable dynamics or trends. |  |
